# Manipulation and Immobilization of a Single Fluorescence Nanosensor for Selective Injection into Cells

**DOI:** 10.3390/s16122041

**Published:** 2016-12-01

**Authors:** Hairulazwan Hashim, Hisataka Maruyama, Taisuke Masuda, Fumihito Arai

**Affiliations:** 1 Department of Micro-Nano Systems Engineering, Nagoya University, Furo-cho, Chikusa-ku, Nagoya, Aichi 464-8603, Japan; hisataka@mech.nagoya-u.ac.jp (H.M.); masuda@mech.nagoya-u.ac.jp (T.M.); arai@mech.nagoya-u.ac.jp (F.A.); 2 Faculty of Engineering Technology, Universiti Tun Hussein Onn Malaysia, 86400 Parit Raja, Batu Pahat, Johor, Malaysia

**Keywords:** nanosensor, fluorescence sensor, photochromism, zeta potential, cell injection

## Abstract

Manipulation and injection of single nanosensors with high cell viability is an emerging field in cell analysis. We propose a new method using fluorescence nanosensors with a glass nanoprobe and optical control of the zeta potential. The nanosensor is fabricated by encapsulating a fluorescence polystyrene nanobead into a lipid layer with 1,3,3-trimethylindolino-6′-nitrobenzopyrylospiran (SP), which is a photochromic material. The nanobead contains iron oxide nanoparticles and a temperature-sensitive fluorescent dye, Rhodamine B. The zeta potential of the nanosensor switches between negative and positive by photo-isomerization of SP with ultraviolet irradiation. The positively-charged nanosensor easily adheres to a negatively-charged glass nanoprobe, is transported to a target cell, and then adheres to the negatively-charged cell membrane. The nanosensor is then injected into the cytoplasm by heating with a near-infrared (NIR) laser. As a demonstration, a single 750 nm nanosensor was picked-up using a glass nanoprobe with optical control of the zeta potential. Then, the nanosensor was transported and immobilized onto a target cell membrane. Finally, it was injected into the cytoplasm using a NIR laser. The success rates of pick-up and cell immobilization of the nanosensor were 75% and 64%, respectively. Cell injection and cell survival rates were 80% and 100%, respectively.

## 1. Introduction

Recently, the manipulation and injection of single nanosensors into cells with minimal invasiveness has received attention due to its potential biological and biomedical applications [1]. For example, measuring the physiological properties of a virus-infected cell is a useful means to investigate the mechanism of viral proliferation to develop new medicines and diagnostic tools [2]. In particular, the investigation of intracellular properties provides useful information [3]. Conventionally, staining whole or part of a cell by fluorescence indicators has been used for intracellular measurement of physiological properties such as temperature, pH, or ion concentrations [4,5,6,7]. Fluorescence measurements are performed by detecting variations in fluorescence intensity and lifetime due to environmental conditions. However, fluorescence measurements have some disadvantages, such as difficulty in controlling the concentration of the indicator and diffusion of the indicator inside cells. Encapsulation of fluorescence indicators into artificial nanobeads allows control over the indicator concentration [8].

Intracellular measurements require selective manipulation and injection of specific nanosensors with minimal invasiveness [9,10]. Several injection methods, such as micro-nano injection [11], endocytosis [12], lipofection [13], electroporation [14], and local heating [15,16] have been developed to inject a particular nanobead into a target cell. In our previous study, we achieved the injection of fluorescence polystyrene nanobeads containing iron oxide nanoparticles by local laser heating [17,18]. The injection times of the fluorescence polystyrene nanobeads are on the order of a few seconds, and survival rates of the injected cells are approximately 100%. However, selective injection with manipulation and cell immobilization of a specific fluorescence polystyrene nanobead has still not been achieved, as the manipulation of a single nanobead in solution is difficult.

Fluorescence polystyrene nanobeads containing iron oxide nanoparticles cannot be manipulated by optical tweezers, since focused laser irradiation onto iron oxide nanoparticles leads to a temperature increase and bubble formation [19]. Electromagnetic tweezers require high current input to the electromagnets to manipulate individual fluorescent polystyrene nanobeads [20,21]. Mechanical manipulation using a glass nanoprobe is suitable for manipulation without environmental fluctuation. However, the success rate for the manipulation of individual fluorescent polystyrene nanobeads using glass nanoprobes with Van der Waals forces is quite low for an unskilled operator.

In this paper, we propose the manipulation and cell immobilization of a single nanosensor using optical control of the zeta potential. We use the Coulomb force for manipulation and immobilization of the nanosensor since the zeta potential of the nanosensor can be controlled using 1,3,3-trimethylindolino-6′-nitrobenzopyrylospiran (SP), which is a photochromic material [22]. The nanosensor is fabricated by staining a polystyrene nanobead containing iron oxide nanoparticles with a temperature-sensitive fluorescence indicator and encapsulating the nanobead into a lipid layer with SP. The zeta potential of the nanosensor, glass, and cell membrane are negative. The zeta potential of the nanosensor becomes positive upon ultraviolet (UV) irradiation due to the photo-isomerization of SP. A positively-charged nanosensor can be picked-up, transported, and immobilized on the cell membrane using the negatively-charged glass nanoprobe. As a demonstration, selective pick-up, transportation, and immobilization on Madin–Darby Canine Kidney (MDCK) cells using optical control of the zeta potential were performed to confirm the effectiveness of the proposed method. Additionally, the cell injection rate and viability of injected cells were evaluated by a fluorescence viability test.

## 2. Materials and Methods

### 2.1. Principle of Manipulation and Cell Injection of the Nanosensor Using Optical Control of the Zeta Potential

**Figure 1:**
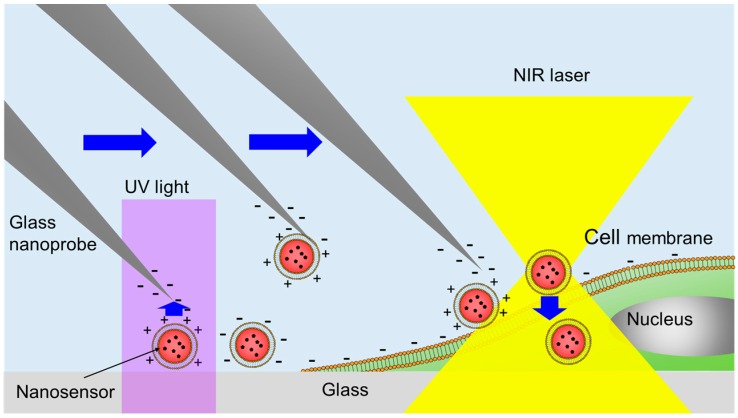
Schematic diagram of the manipulation and cell injection of a single nanosensor using optical control of the zeta potential. NIR: Near-infrared.

Figure 1 shows the principle of the manipulation and cell injection of a single nanosensor to a target cell using optical control of the zeta potential. The nanosensor is made up of a polystyrene nanobead containing iron oxide nanoparticles, Rhodamine B, a lipid layer, and SP. Rhodamine B is a temperature-sensitive fluorescence indicator. The lipid layer is composed of the neutral lipid dioleoylphosphocholine (DOPC). SP, purchased from Tokyo Chemical Industry Co. Ltd. (Tokyo, Japan), is used for zeta potential control. The nanosensor is fabricated by staining the nanobead with Rhodamine B and encapsulating the stained nanobead by the lipid layer with SP. Zeta potentials of the glass nanoprobe and polystyrene nanobead in water are approximately −50 mV and −30 mV, respectively [23]. DOPC is electrically neutral. The molecular conformation of SP changes from the *cis*-type to *trans*-type by UV irradiation, and is recovered by visible (VIS) light irradiation. The zeta potential of the nanosensor switches from negative to positive by UV irradiation, since the zeta potential of *trans*-type SP is higher than that of *cis*-type SP. Therefore, the positively-charged nanosensor adheres to the glass nanoprobe and cell membrane.

First, the selected nanosensor is irradiated with UV light to switch the zeta potential from negative to positive. Then, the positively-charged nanosensor is picked-up and transported to the target cell using the glass nanoprobe due to the attractive Coulomb force. Negatively-charged nanosensors do not adhere to the glass nanoprobe because of the repulsive force. The transported nanosensor is immobilized on a cell membrane. For example, the zeta potential of an MDCK cell is approximately –40 mV [24]. The positively-charged nanosensor can immobilize on the cell membrane and detach from the glass nanoprobe by pushing the cell membrane with the nanosensor to increase the contact area. The zeta potentials of the glass nanoprobe and MDCK cells are similar. Therefore, an increase in contact area between the nanosensor and cell is needed to detach the nanosensor from the glass. Flexibility of the glass nanoprobe is also useful for manipulation of the nanosensor on the cell membrane without damage to the cell.

After cell immobilization, the nanosensor is injected into cell cytoplasm by local laser heating. In this study, cell activities such as endocytosis and lipofection were not used for injection because the injection of large nanoparticles by either method takes a long time. In our previous research, cell injection of the nanosensor made up of a 750 nm polystyrene nanobead containing iron oxide nanoparticles using laser heating (wavelength: 1064 nm, power: 28 mW) was achieved within a few seconds [17]. The iron oxide absorbs the 1064 nm light and generates heat locally. We used the same polystyrene nanobeads as material for the nanosensors, and the same laser wavelength and power for cell injection in the present study.

### 2.2. Experimental System Setup

**Figure 2:**
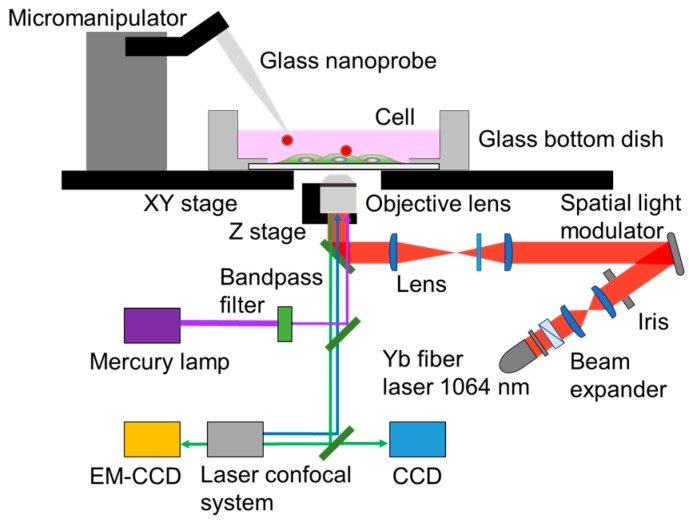
A schematic diagram of the experimental system. CCD: Charge-coupled device; EM-CCD: Electron multiplying-CCD.

Figure 2 shows a schematic diagram of the experimental setup consisting of optical and fluorescence microscopy systems. An inverted optical microscope (IX71, Olympus, Tokyo, Japan) having an epi-fluorescence observation system and laser confocal system was used to observe the nanosensor and cell. A 3-degrees of freedom (DOF) micromanipulator (SMX, Sensapex, Oulu, Finland) was used for manipulation of the nanosensor. The range of motion for all three dimensions was 20 mm, and the step resolution was 30 nm. To observe the manipulation process, we used a digital charge-coupled device (CCD) camera (Grasshopper, Point Gray, Richmond, BC, Canada). A confocal laser scanning system (CSU-X1, Yokogawa Electric Co., Tokyo, Japan) with an excitation laser of 488 nm and 561 nm, and electron multiplying-CCD (EM-CCD) camera (DU-897, iXon, Andor Technology Ltd., Belfast, UK) was used to acquire fluorescence images. The movement of the piezoelectric *z*-stage (E-665, Physik Instrument GmbH & Co. KG, Karlshure, Germany) that coupled to the high-magnification objective lens (UPlanSApo 100×/1.40, Olympus, Tokyo, Japan) was used to acquire 3D fluorescence images. A mercury lamp was used for photo-isomerization of SP by UV irradiation. A 1064 nm near-infrared (NIR) laser with a maximum power of 10 W was used for local heating [25]. The beam diameter at the focus was 1.4 μm. In this study, the power of the NIR laser was adjusted to 28 mW [17].

### 2.3. Optical Control of Zeta Potential Using Photochromic Material

**Figure 3:**
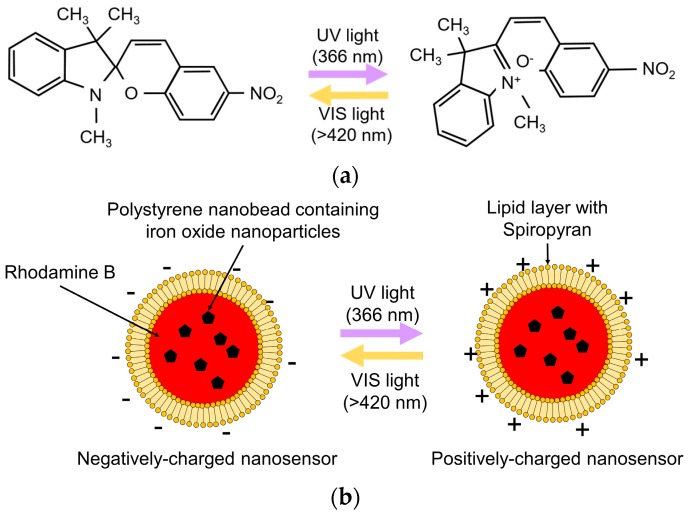
Schematic diagram of optical control of zeta potential. (**a**) Changes in the molecular structure of SP by photo-isomerization; (**b**) Optical control of zeta potential of the nanosensor.

Figure 3 shows a schematic diagram of the optical control of the zeta potential of the nanosensor using SP. The molecular structure of SP changes from *cis*-type (left side) to *trans*-type (right side) by UV irradiation. The zeta potential of the *trans*-type structure is higher than that of the *cis*-type structure [26]. This photo-isomerization is reversible and repeatable by UV/VIS irradiation. A schematic diagram of the nanosensor is shown in Figure 3b. The polystyrene bead containing iron oxide nanoparticles is encapsulated by a lipid layer with SP. The zeta potential of the nanosensor changes from negative to positive upon UV irradiation. The positively charged nanosensor adheres to the negatively charged glass nanoprobe and cell membrane.

### 2.4. Fabrication of the Nanosensor with a Photochromic Lipid Layer

**Figure 4:**
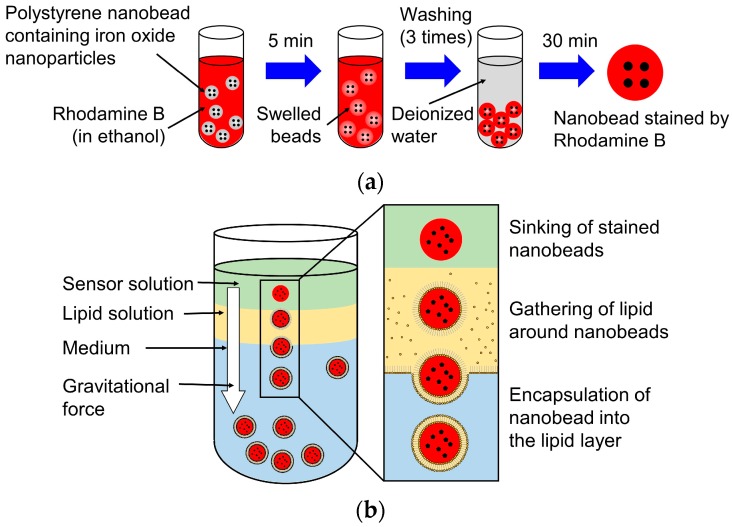
Fabrication process of the nanosensor. (**a**) Stain of the polystyrene nanobead by hodamine B; (**b**) Encapsulation of the fluorescence polystyrene nanobead into the lipid layer with 1,3,3-trimethylindolino-6′-nitrobenzopyrylospiran (SP).

**Figure 5:**
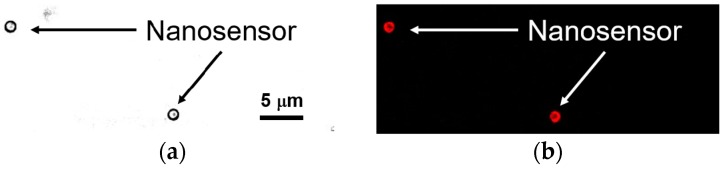
Optical and fluorescence image of the nanosensor. (**a**) Optical image; (**b**) Fluorescence image.

The nanosensor was made up of 750 nm polystyrene nanobeads containing iron oxide nanoparticles (EPRUI Nanoparticles & Microspheres Co. Ltd., Nanjing, China), Rhodamine B, DOPC, and SP. The diameter of iron oxide nanoparticles inside the polystyrene nanobead ranges from 20 to 40 nm [17]. Figure 4 shows the fabrication process of the nanosensor. First, polystyrene nanobeads containing iron oxide nanoparticles were stained with 6 g/L Rhodamine B in ethanol, as shown in Figure 4a. Rhodamine B is a temperature-sensitive fluorescence indicator. After immersion in ethanol for 5 min, the stained polystyrene nanobeads were washed with deionized (DI) water three times. Then, the fluorescence polystyrene nanobeads were encapsulated in the lipid layer with SP by spontaneous transport [27,28], as shown in Figure 4b. DOPC, a non-charged lipid, was used to form the lipid layer. The lipid layer was prepared by mixing 10 mM of DOPC and 40 μM of SP in mineral oil. After forming a multilayer of 0.7 mL of phosphate buffered saline (PBS) solution and 0.3 mL of lipid solution in a microtube, a 0.2 mL mixture of the fluorescence polystyrene nanobeads and mineral oil was introduced. During the sinking of the fluorescent polystyrene nanobeads by gravity, the lipids gathered and formed a layer with SP on the surface of the nanosensor. Figure 5 shows optical and fluorescence images of fabricated nanosensors. The nanosensors were excited by the 561 nm laser in the fluorescence image. The mean diameter of the nanosensors was 1089 nm, which was evaluated by a tunable resistive pulse sensing (TRPS) nanoparticle analyzer (qNano, Izon Science Ltd., Christchurch, New Zealand). The thickness of the lipid layer was also measured to be approximately 170 nm. The concentration of the nanosensor after fabrication was 1.4 × 10^10^ particles/mL. The nanosensor was diluted to a suitable concentration in the experiments.

### 2.5. Fabrication of the Glass Nanoprobe

A glass nanoprobe was used for manipulation of the nanosensor and was controlled by a 3-DOF micromanipulator. The nanoprobe was fabricated by a borosilicate glass rod (G-1000, Narishige Scientific Instrument Lab., Tokyo, Japan) and then pulled using a magnetic glass microelectrode horizontal puller (PN-31, Narishige Scientific Instrument Lab., Tokyo, Japan). The tip diameter of the glass nanoprobe was less than 1 μm.

### 2.6. Cell Culture

MDCK cells were used for the experiments in this study. MDCK cells were cultured in a glass-bottom dish with 2.7 mL of Dulbecco’s Modified Eagle Medium (DMEM) and 0.3 mL of fetal bovine serum (FBS). The culture conditions were an atmosphere of 5% CO_2_ and 95% air at 37 °C temperature. MDCK cells were cultured for 8 h before the experiments. The cells were stained with Calcein-AM to test for cell viability after injection. To dye the cell membranes, the cells were washed twice using PBS, then 10 μL of 0.5 mg/mL Calcein-AM solution was mixed with 5 mL of PBS to produce the dye solution. Then, 1 mL of culture medium in the dish was replaced with the dye solution. After 30 min of incubation at 37 °C, the stained MDCK cells were used for the experiments.

## 3. Results and Discussion

In this study, we first calibrated the temperature with relative fluorescence intensity of the nanosensor. Then, pick-up and cell immobilization of the nanosensor by optical control of the zeta potential was performed, and the success rates without and with zeta potential control were compared. Finally, injection of the immobilized nanosensor by local heating was performed, and the success rates of injection and viability of the injected cells were evaluated.

### 3.1. Temperature Calibration of the Nanosensor 

**Figure 6:**
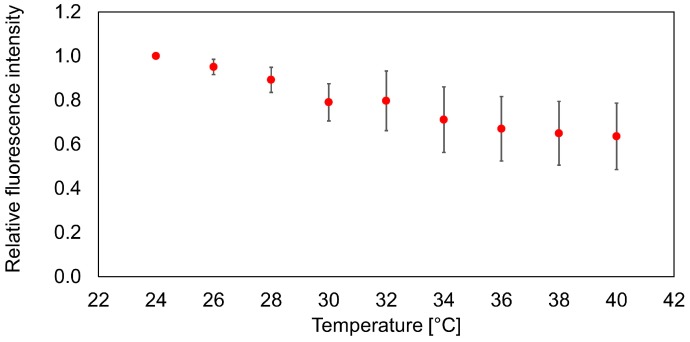
Measurement of fluorescence intensity vs. temperature to construct a calibration curve. The error bars represent the standard deviation of average fluorescence intensity from nine stained nanosensors.

In this study, we fabricated nanosensors for temperature measurements. The principle of the temperature measurement is measurement of the variation in fluorescence intensity of a nanosensor due to changes in temperature. The fluorescence intensity of Rhodamine B decreases according to the temperature increase [8]. Figure 6 shows a calibration curve of the relative fluorescence intensity vs. temperature based on 24 °C. The environmental temperature was controlled by a cell culture chamber (ZILCS, Tokai Hit. Co. Ltd. Shizuoka, Japan) with an accuracy of 0.3 °C. The calibration temperature ranged from 24 °C to 40 °C. From this calibration curve, the sensitivity of the nanosensor was determined to be –2.4%/°C.

### 3.2. Manipulation and Immobilization of the Nanosensor Using Zeta Potential Control

**Figure 7:**
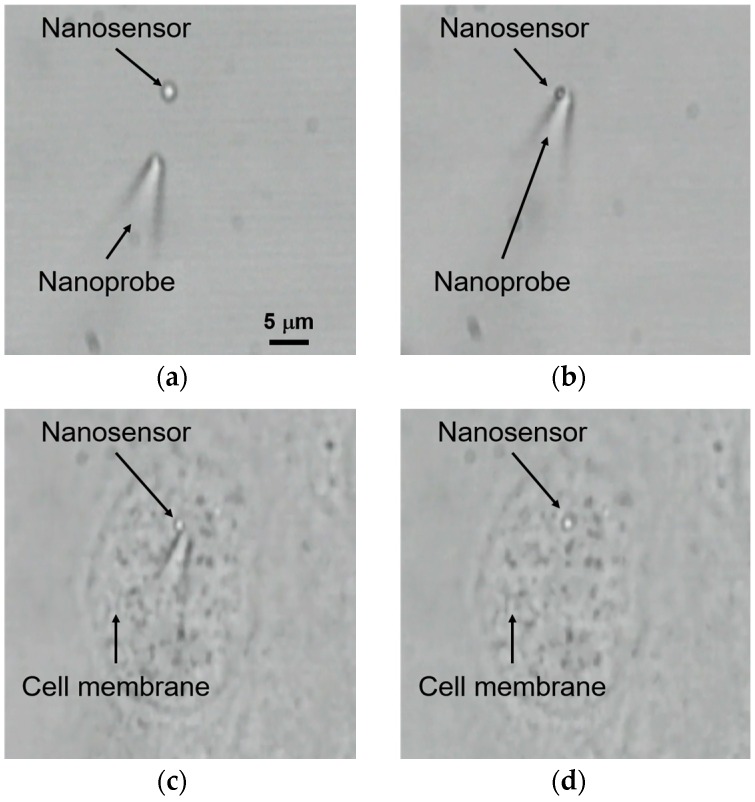
Pick-up and immobilization of the nanosensor using a micromanipulator and optical control of the zeta potential. (**a**) Approach of the glass nanoprobe under UV irradiation; (**b**) Pick-up of the nanosensor; (**c**) Contact of the nanosensor to the membrane of an Madin–Darby Canine Kidney (MDCK) cell; (**d**) After immobilization of the nanosensor.

Figure 7 shows experimental results of pick-up and immobilization of the nanosensor using a micromanipulator and zeta potential control. The final concentration of the nanosensor in the glass bottom dish was 9.3 × 10^7^ particles/mL. A single nanosensor could be picked-up within a few minutes at this concentration. At first, UV light from the mercury lamp (λ: 330–380 nm) was used to irradiate the target nanosensor. The UV and VIS power densities were about 3.5 mW/cm^2^ and 5.4 mW/cm^2^, respectively. The zeta potential of the UV-irradiated nanosensor switched to positive. The nanosensor was picked up using a glass nanoprobe by the Coulomb force, as shown in Figure 7b. The nanosensor was kept on the glass nanoprobe for at least 5 min under VIS light irradiation. Table 1 shows the success rate of pick-up of the single nanosensor with and without UV irradiation in a different solution. The effect of the solution was evaluated using PBS and DMEM + FBS. Over ten nanosensors were evaluated under each condition. In PBS, the success rate of the pick-up of the nanosensor without UV irradiation was only 10%. On the other hand, the success rate was increased to 75% with UV irradiation. In DMEM + FBS, the success rates of pick-up with and without UV irradiation were 43% and 5%, respectively. Based on these results, we concluded that PBS is suitable for pick-up of the nanosensor using optical control of the zeta potential.

Figure 7c,d shows immobilization of the nanosensor to an MDCK cell. The positively-charged nanosensor on the glass nanoprobe contacted the cell membrane and was immobilized on it by the Coulomb force, since the zeta potential of the cell was negative. The success rate of cell immobilization with UV irradiation was 64%. The success rate of cell immobilization was lower than that of pick-up. The reason is likely the effect of the position of the nanosensor on the glass nanoprobe. When the nanosensor was on the tip of the glass nanoprobe, the success rate of cell immobilization was high, since contact of the nanosensor with the cell membrane is easy. On the other hand, when the nanosensor was at the upper side of the glass nanoprobe, the success rate was low, since contact with the cell membrane is difficult. This problem will be addressed by adding a rotating mechanism to the micromanipulator for attitude control of the nanosensor.

### 3.3. Injection of the Nanosensor by Local Laser Heating

**Figure 8:**
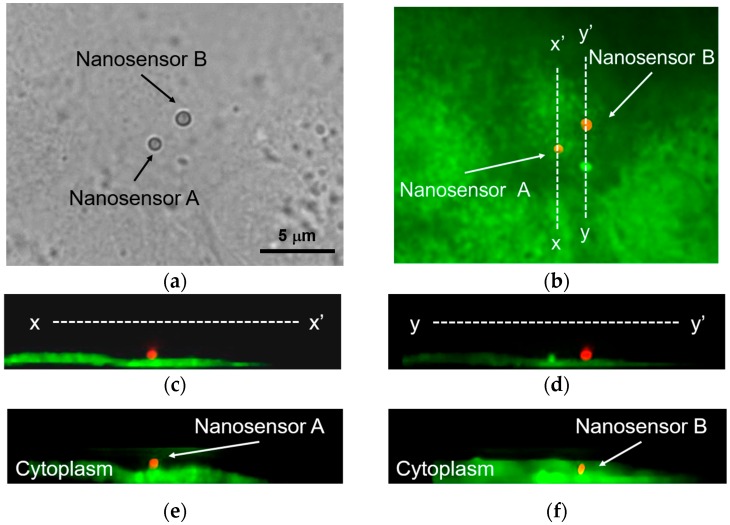
Injection of the immobilized sensor by local laser heating. (**a**) Optical image of the nanosensors on an MDCK cell; (**b**) Fluorescence image before injection (red is the nanosensor, bright green is fluorescence from the cell); (**c**) Cross-sectional view of nanosensor A before laser irradiation (x-x’); (**d**) Cross-sectional view of nanosensor B before laser irradiation (y-y’); (**e**) Cross-sectional view of nanosensor A after laser irradiation; (**f**) Cross-sectional view of nanosensor B after laser irradiation.

Figure 8 shows the injection result of the nanosensor into an MDCK cell by local laser heating. MDCK cells were stained with a cell-permeable fluorescent dye (Calcein-AM) to observe the inside of the cells and test their viability. Calcein-AM passes through the cell membrane and is hydrolyzed by esterase activity in a living cell. Hydrolyzed Calcein-AM emits green fluorescence. Local laser heating was performed using the focused NIR laser at 28 mW, as in our previous research [17]. The extinction coefficient of water is 14.2 m^−1^ at 1064 nm [25]. This value is quite small, so the temperature increase of water by laser heating can be ignored.

Figure 8a shows the optical image of two nanosensors on an MDCK cell. Positions of nanosensors A and B were confirmed by cross-sectional fluorescence imaging, as shown in Figure 8c,d. In these images, both nanosensors are red, and the MDCK cell is green. Therefore, both sensors were located on the cell membrane. The distance between these nanosensors was approximately 3 μm. After irradiation with the NIR laser to nanosensor B for one second, it was injected into the MDCK cell. The positions of these nanosensors are shown in Figure 8e,f. While nanosensor B was inside the MDCK cell, nanosensor A was still on the cell membrane. This result indicates that we succeeded in the local injection of an arbitrary nanosensor on the cell membrane by local laser heating. Moreover, the MDCK cell injected with the nanosensor still emitted green fluorescence. Among five attempts to inject a nanosensor using local laser heating, four were successfully injected, and all four cells were alive after injection. Therefore, the success rate of injection and cell viability were 80% and 100%, respectively. The effect of the nanosensor size was not examined in this study. We previously reported the injection of polystyrene nanobeads containing iron oxide nanoparticles by local laser heating [17,18]. The diameters of the polystyrene nanobeads were 300 nm and 750 nm. As in that study, the polystyrene nanobeads used here had a diameter of 750 nm. Applying the current method to other bead sizes will be the topic of a future study.

## 4. Conclusions

We have achieved a new approach for the manipulation and cell injection of a single nanosensor into a cell using a glass nanoprobe with optical control of the zeta potential and local laser heating. This method was suitable for selective manipulation of a fluorescence nanosensor, since the tip was safe for contact with cell membranes, and transparency of the glass allowed for observation of the nanosensor. This approach was also suitable for improving immobilization of the nanosensor to the cell membrane. The nanosensor was stained with Rhodamine B, and could be used for temperature measurement. Photoisomerization of SP achieved a switch of the zeta potential of the nanosensor from negative to positive. The positively-charged nanosensor made the pick-up and transport by the negatively-charged glass nanoprobe and immobilization on a cell membrane much easier rather than without UV irradiation. Pick-up and cell immobilization of the nanosensor were improved to 75% and 64%, respectively. Moreover, the immobilized nanosensor was injected into the cell cytoplasm by NIR laser irradiation within one second. The injection rate and viability of the injected cells were 80% and 100%, respectively.

In our previous study, our group achieved pH measurement of an influenza virus-infected cell on the cell membrane using a fluorescence microsensor [29]. However, intracellular measurement was not achieved, since rapid injection of the selected microsensor into a specific cell was still too difficult. The current proposed method allowing the injection of a fluorescence nanosensor will be a break-through for single cell analyses such as pH and temperature measurements inside virus-infected cells.

## Figures and Tables

**Table 1:** Success rate of pick-up of the nanosensor by the glass nanoprobe without/with UV irradiation. DMEM: Dulbecco’s Modified Eagle Medium; FBS: Fetal bovine serum; PBS: Phosphate-buffered saline.

Solution	Without UV	With UV
PBS	10%	75%
DMEM + FBS	5%	43%
